# Investigation of *GSDME* results in the identification of the first pathogenic synonymous variants and genotype-phenotype correlations

**DOI:** 10.1007/s00439-025-02782-6

**Published:** 2025-09-29

**Authors:** Joseph J. Chin, W. Daniel Walls, Kai Wang, Amanda M. Odell, Diana L. Kolbe, Kevin T. A. Booth, Hela Azaiez, Richard J. H. Smith

**Affiliations:** 1 Medical Scientist Training Program, Carver College of Medicine, University of Iowa, 375 Newton Road 2133 Medical Laboratories, Iowa City, IA 52242 USA; 2 Molecular Otolaryngology and Renal Research Laboratories, Carver College of Medicine, University of Iowa, 200 Hawkins Drive—21151 PFP, Iowa City, IA 52242 USA; 3 Department of Biostatistics, College of Public Health, University of Iowa, 145 N. Riverside Drive, IA 52242 Iowa City, USA; 4 Department of Medical and Molecular Genetics, Indiana School of Medicine, 975 W. Walnut Street, IN 46202 Indianapolis, USA; 5 Department of Otolaryngology, Indiana School of Medicine, 1130 W. Michigan Street, IN 46202 Indianapolis, USA

## Abstract

Despite advances in the genetic diagnosis of hearing loss, there remains room for improvement. One way to improve the genetic diagnostic rate is the proper assessment of synonymous variants that are often bioinformatically filtered out. We used *GSDME* as a model to demonstrate the importance of assessing synonymous variants. Variants in the gene *GSDME* (also known as *DFNA5*) are associated with autosomal dominant nonsyndromic hearing loss. The hearing loss is typically progressive and downsloping. All reported causative variants of *GSDME*-related hearing loss involve the skipping of exon 8, which results in the expression of a constitutively active, but truncated protein that induces apoptosis of cochlear hair cells. A retrospective search of previously tested patients identified 3 novel pathogenic synonymous *GSDME* variants. The functional impact of these variants was confirmed in vitro via a minigene splicing assay. We also observed variant-dependent differences in the levels of aberrant splicing, leading us to hypothesize that partial loss of splicing will result in a less severe hearing loss phenotype as compared to complete loss of splicing. Audiometric analysis found an association between complete loss of splicing and greater initial and/or more quickly progressing hearing loss as compared to partial loss of splicing. Over the course of the study, we also found limited correlation between in silico prediction and in vitro observed effects of a variant on splicing, indicating the need to cautiously apply in silico prediction tools in the context of genetic diagnosis.

**Supplementary Information:**

The online version contains supplementary material available at 10.1007/s00439-025-02782-6.

## Introduction

The clinical care of the deaf/hard-of-hearing is an important component of healthcare. Hearing loss (HL) is the most common sensory deficit, affecting 1 in 500 newborns (Fortnum et al. 2001; Vohr 2003; Morton and Nance 2006; Centers for Disease Control and Prevention (CDC) 2010) and up to 50% of octogenarians (Lin et al. 2011). Over the past decade, the diagnostic algorithm for these patients has changed dramatically. Comprehensive genetic testing is the foundational diagnostic test (Alford et al. 2014) because it is cost-effective (Jayawardena et al. 2015; Sloan-Heggen and Smith 2016) and enables health-care providers to make evidence-based decisions (Sloan-Heggen et al. 2016) that reduce overall healthcare costs (Eppsteiner et al. 2012; Miyagawa et al. 2013; Nishio and Usami 2017; Boudewyns et al. 2018). Currently, the underlying genetic basis for HL can be diagnosed in ~ 40% of cases, but it can be as high as ~ 72% (Sloan-Heggen et al. 2016) in well studied populations. This disparity in diagnostic rate indicates potential for significant improvement. Given that ten genes account for over 70% of the diagnoses (Sloan-Heggen et al. 2016), the source of missed diagnoses is likely a combination of variants within common known HL genes and yet-to-be discovered rare HL genes (Kremer 2022). This gap between the presumed prevalence of a Mendelian condition and the rate of a precise genetic diagnosis has been seen in many other genetic disorders (Wojcik et al. 2023).

In the case of HL, missing diagnoses may occur due to unappreciated mechanisms of pathogenesis. Of particular interest are splice-altering synonymous variants. Synonymous variants are often filtered out in bioinformatics workflows under the assumption that they are benign (Hirsch et al. 2021; Wang et al. 2023), despite being a major source of pathogenic variants. Examples of pathogenic splice-altering synonymous variants have been observed in both hearing (Hirsch et al. 2021) and non-hearing genetic disorders (Eriksson et al. 2003). As a result, filtering out presumed synonymous variants can be detrimental to obtaining a genetic diagnosis. Given that synonymous variants contribute to 28% of coding variants in known HL associated genes (Azaiez et al. 2018), we hypothesized that there are many yet to be discovered pathogenic synonymous variants.

We used *GSDME* as a model to demonstrate the importance of assessing synonymous variants as potentially disease-causing because all known pathogenic *GSDME* variants involve the formation of a transcript that lacks exon 8 and thereby encodes a constitutively active, truncated protein (de Beeck et al. 2012). This eliminates the need to consider other pathologies linked to synonymous variants and allows us to focus the assessment of synonymous variants on just the impact on splicing. Our investigation of *GSDME* identified 3 novel pathogenic synonymous variants and variant-dependent differences in the level of aberrant splicing. This variability in aberrant splicing led us to hypothesize that partial loss of splicing may result in a milder HL phenotype as compared to complete loss of splicing. We also sought to determine whether in silico prediction tools can be used to rapidly screen for pathogenic *GSDME* variants.

## Materials and methods

### Subjects

All procedures were approved by the human research Institutional Review Board at the University of Iowa, Iowa City, Iowa (USA). When possible, we attempted to obtain DNA samples and audiograms from other family members with HL.

Persons of interest were identified in a retrospective search of a cohort of patients previously tested by the Molecular Otolaryngology and Renal Research Laboratories (MORL). The cohort consisted of approximately 7,450 patients with HL and comprehensive testing for genetic HL (OtoSCOPE^®^). Existing sequencing information was searched to identify rare synonymous *GSDME* variants in the genomic coordinates of exon 8 for in vitro functional assessment (meeting the strictest criteria for PM2_Sup by ACMG criteria (Oza et al. 2018) and https://clinicalgenome.org/site/assets/files/5182/pm2_-_svi_recommendation_-_approved_sept2020.pdf. Rare synonymous *GSDME* variants were included regardless of the reported family history, clinical correlation, and in silico prediction. These rare synonymous variants were also used as a retrospective cohort for the assessment of in silico prediction tools.

### Next-Generation sequencing and bioinformatic analysis

All probands in the retrospective MORL cohort had prior targeted genomic enrichment and massively parallel sequencing via the OtoSCOPE^®^ platform. Probands carrying a synonymous *GSDME* variant of interest were reanalyzed using an updated version of the bioinformatic analysis as previously described (Shearer et al. 2010; Sloan-Heggen et al. 2016; Booth et al. 2018), with modifications to include the reporting and assessment of predicted synonymous variants. All *GSDME* variants were aligned to the NM_004403.3 transcript. Probands carrying a *GSDME* variant that had functional consequences when assessed in vitro were discussed in further detail at an interdisciplinary Hearing Group Meeting that included clinicians, scientists, and genetic counselors to evaluate all high quality variants with a minor allele frequency < 1% with ACMG criteria (Alford et al. 2014; Oza et al. 2018). Information regarding the variants in other genes and population specific data regarding the *GSDME* variants is available in Supplementary Tables 1 and 2.

### Segregation analysis

Segregation analysis was performed by Sanger sequencing on an Applied Biosystems 3500 Series Genetic Analyzer (Waltham, MA). Chromatograms were visualized using Sequencher^®^ v5 (Gene Code Corporation, Ann Arbor, MI). Pedigrees of reported family history were generated using QuickPed (Vigeland 2022).

### In vitro splicing analysis

The effects of the variant were assessed in vitro via minigene splicing assay as previously described (Booth et al. 2018) with some modifications. Briefly, the preconstructed pET01 Exontrap vector (MoBiTec) was linearized using the SalI-HF^®^ and SacII^®^ (New England Biolabs) restriction enzymes. A wild-type *GSDME* fragment that included *GSDME* exon 8 and 186 and 200 nucleotides from the 3′ and 5′ flanking sequences was generated using PCR (NEB Q5^®^ High-Fidelity DNA Polymerase). The resulting fragment was inserted and joined with the linearized pET01 fragment using the NEBuilder^®^ HiFi kit. The relevant mutation was introduced into the wild-type fragment using the Q5^®^ Site-Directed Mutagenesis kit (New England Biolabs). The inserts in all constructs were Sanger sequenced to verify their identity and presence of the correct mutation.

Wild-type, mutant, and empty vectors were transfected into COS-7 cells in triplicate using Lipofectamine™ LTX with Plus™ Reagent (Thermo Fisher Scientific). Cells were harvested 36–48 h post transfection. RNA was isolated using the RNeasy^®^ Plus Mini Kit (Qiagen). cDNA was transcribed using 500 ng of isolated total RNA, RNA SuperScript™ III Reverse Transcriptase (Thermo Fisher Scientific), and a primer specific to the 3′ native exon of the pET01 vector. PCR amplification was performed using primers specific to the 5′ and 3′ native exons of the pET01 vector. Products were visualized on a 1.5% agarose gel.

Because we identified cases of partial (“leaky”) loss of splicing in the newly identified synonymous variants, we used multiple positive controls to confirm that partial loss of splicing is sufficient to cause HL. The previously identified pathogenic c.991–2 A > G, c.1102 C > G, c.1154 C > T, and c.1183G > A variants were used as positive controls. The benign variant c.1122 C > T (rs138980048:G > A) was used as a negative control.

### Audiometric profiling

Audiometric profiling was used to determine if there was a difference in the age of onset and/or rate of progression of HL between variants that result in complete loss of splicing versus partial loss of splicing. A literature review (PubMed search terms GSDME OR DFNA5) and the Deafness Variation Database (Azaiez et al. 2018) (accessed April 2024) was performed to identify *GSDME* variants where there was information regarding the amount of splicing lost. Audiograms were collected from the AudioGene v4.0 database, the literature review, and the MORL’s patient database. In the event there were data from both ears, the better hearing ear was used at each frequency. Because differences in methods used to quantify the loss of splicing made it impractical to make exact comparisons of the amount of splicing lost, we chose to focus on three variants with an extensive audiometric history and used a qualitative classification of complete versus partial loss of splicing (Supplementary Table 3).

Since many of the audiograms were taken from the same individual longitudinally, audioprofiling was conducted by fitting linear regression lines using a Tobit (censored) mixed effects model to account for individual differences and censoring of HL thresholds due to audiometer limits. Comparisons were made to identify differences in audioprofiles between partial versus complete loss of splicing, as well as for variant-specific effects. Upper and lower censoring thresholds were set at 0 and 110 dB respectively, to account for differences in minimum and maximum outputs of audiometers (https://www.asha.org/policy/GL2005-00014/). Bonferroni correction was applied for multiple comparisons. These regressions were then used to generate Age-Related Typical Audiograms (ARTAs) in 10-year age bins. Regressions were performed in R (version 4.5.0; (R Core Team 2023) using the censReg (version 0.5–38; (Henningsen 2024) and lme4 (version 1.1–37; (Bates et al. 2015) packages.

### Analysis of in silico prediction tools

We sought to determine whether it was feasible to use in silico prediction tools to rapidly screen for splice-altering variants. Since splice-altering variants have been reported throughout *GSDME* exon 8 and the flanking intronic regions (Booth et al. 2018), we focused on testing Human Splicing Finder v3.1 (HSF) (Desmet et al. 2009), SpliceAI (Jaganathan et al. 2019), and Splicing Prediction Pipeline (SPiP) (Leman et al. 2022) because of their ability to assess variants regardless of their proximity to the splice donor and acceptor regions. SpliceAI scores were obtained via the Ensembl Variant Effect Predictor plugin (McLaren et al. 2016), run on the Ensembl release 110 and the GRCh37.p13 assembly. The recommended SpliceAI threshold of at least 0.5 (Jaganathan et al. 2019) was used as a cutoff for a predicted splice-altering effect.

We also sought to determine whether conservation can be used to screen for potential pathogenic variants. The correlation between conservation and impact on splicing was determined by extracting the 100 vertebrates basewise PhyloP conservation scores from the UCSC Genome Browser and generating ROC curves in GraphPad Prism version 10.4.1 (San Diego, CA). This analysis was done in two separate cohorts consisting of the variants found from the MORL patient cohort and from gnomAD.

Variants from the gnomAD cohort were obtained by identifying rare (PM2_Supporting for autosomal dominant nonsyndromic HL (ADNSHL) by ACMG criteria (Oza et al. 2018) synonymous variants in *GSDME* exon 8 from gnomAD v2.1.1 (Karczewski et al. 2020). These variants were included regardless of whether they had a positive or negative in silico prediction. Using this strategy, we identified 14 rare synonymous variants in gnomAD to assess in a minigene splicing assay. These 14 variants include the c.1008 C > T, p.(Ser336Ser) variant, which we independently identified during our retrospective review of the MORL patient database. We also performed a retrospective analysis of 11 previously reported pathogenic SNVs and indels (Booth et al. 2018; Azaiez et al. 2018).

## Results

### Identification of three pathogenic synonymous variants in the MORL patient cohort

**Fig. 1:**
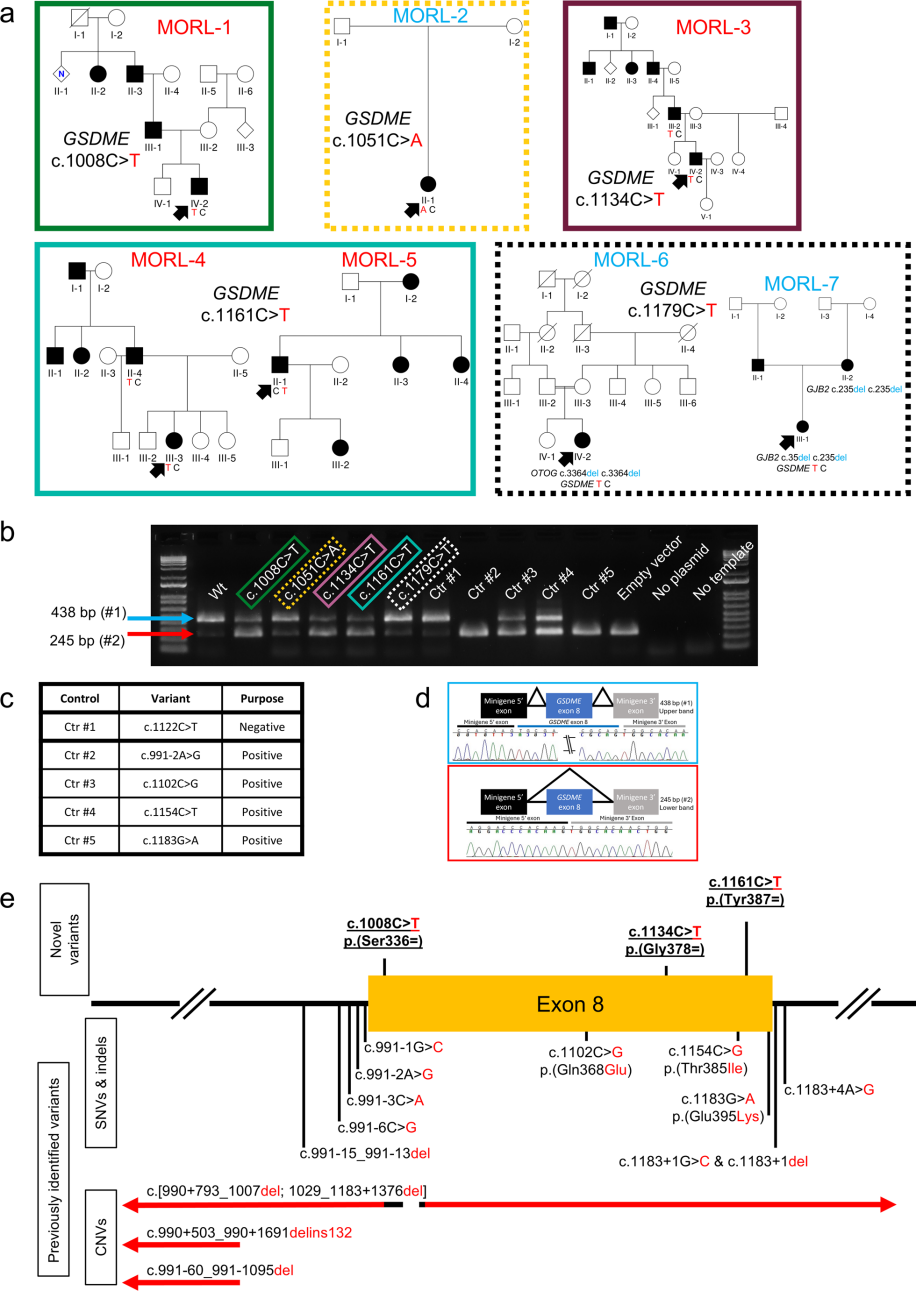
Identification of pathogenic synonymous variants. **a** Pedigrees showing the reported family history and available segregation analysis of persons carrying rare synonymous *GSDME* variants in the MORL cohort. Probands who underwent OtoSCOPE^®^ testing are indicated with an arrow. Families in red font and surrounded by solid boxes have *GSDME* variants that resulted in altered splicing. Families in blue font and surrounded by dashed boxes did not have splice-altering *GSDME* variants. **b** Minigene splicing assay results visualized on gel electrophoresis for wild-type and mutant constructs. Five synonymous variants were analyzed. The c.1008 C > T, c.1134 C > T, and c.1161 C > T synonymous variants, along with two of the four previously reported variants, result in partial loss of splicing, as indicated by the presence of both an upper band (438 bp, #1) and a lower band (245 bp, #2). **c** Table describing the controls, their identity, and function. **d** Schematic representation and sequencing of the upper band (containing exon 8) and the lower band (lacking exon 8). **e** Schematic of known pathogenic *GSDME* variants and their relative location in *GSDME* exon 8. Novel variants are depicted at the top, while previously reported variants are shown at the bottom

A retrospective analysis of the MORL cohort identified five synonymous variants in *GSDME* exon 8 for functional assessment. These variants were observed in 7 unrelated probands (Fig. 1a). Three of the five synonymous variants were shown to be splice-altering in a minigene splicing assay (Fig. 1b). The loss of splicing for these three novel pathogenic synonymous variants was shown to be a partial loss of splicing, as demonstrated by the presence of both an upper band (with exon 8) and a lower band (lacking exon 8). This partial loss of splicing in the synonymous variants is consistent with the results seen in two of four of the previously reported variants used as positive controls (Fig. 1b-d and Supplementary Table 4).

All three splice-altering variants were linked to families with a reported family history consistent with an autosomal dominant inheritance pattern (Fig. 1a). Reported physical exams did not note any syndromic features. None of the patients reported any signs of vestibular dysfunction or delayed motor milestones. The c.1008 C > T variant was found in family MORL-1, which has a reported paternal family history of HL. Audiograms were unavailable from this patient, but the patient reported a clinical diagnosis of HL at 13 years old. The proband also has a *MT-RNR1* m.1494 C > T variant (Supplementary Table 1), which has a high level of evidence linking it to aminoglycoside-inducted HL (McDermott et al. 2022). There was no available information on the proband’s exposure to aminoglycosides. While this may raise the possibility of a dual diagnosis, we highlight that the *MT-RNR1* variant does not explain the paternal inheritance pattern in the family MORL-1. This potential dual diagnosis demonstrates the need to collect and analyze genetic sequencing data in the context of detailed clinical information. The c.1134 C > T variant was found in family MORL-3, which has a four-generation history of HL. The c.1161 C > T variant was found in two families, MORL-4 and MORL-5. Available audioprofiles and segregation data for carriers of the c.1134 C > T and c.1161 C > T variants were consistent with *GSDME*-related HL (Supplementary Figs. S1 and S2). The two *GSDME* variants that did not alter splicing (c.1051 C > A and c.1179 C > T) were found in families (MORL-2, MORL-6, and MORL-7) that had a reported family history inconsistent with ADNSHL and/or an alternative genetic diagnosis (Fig. 1a, Supplementary Table 1).

### Dataset composition

**Fig. 2:**
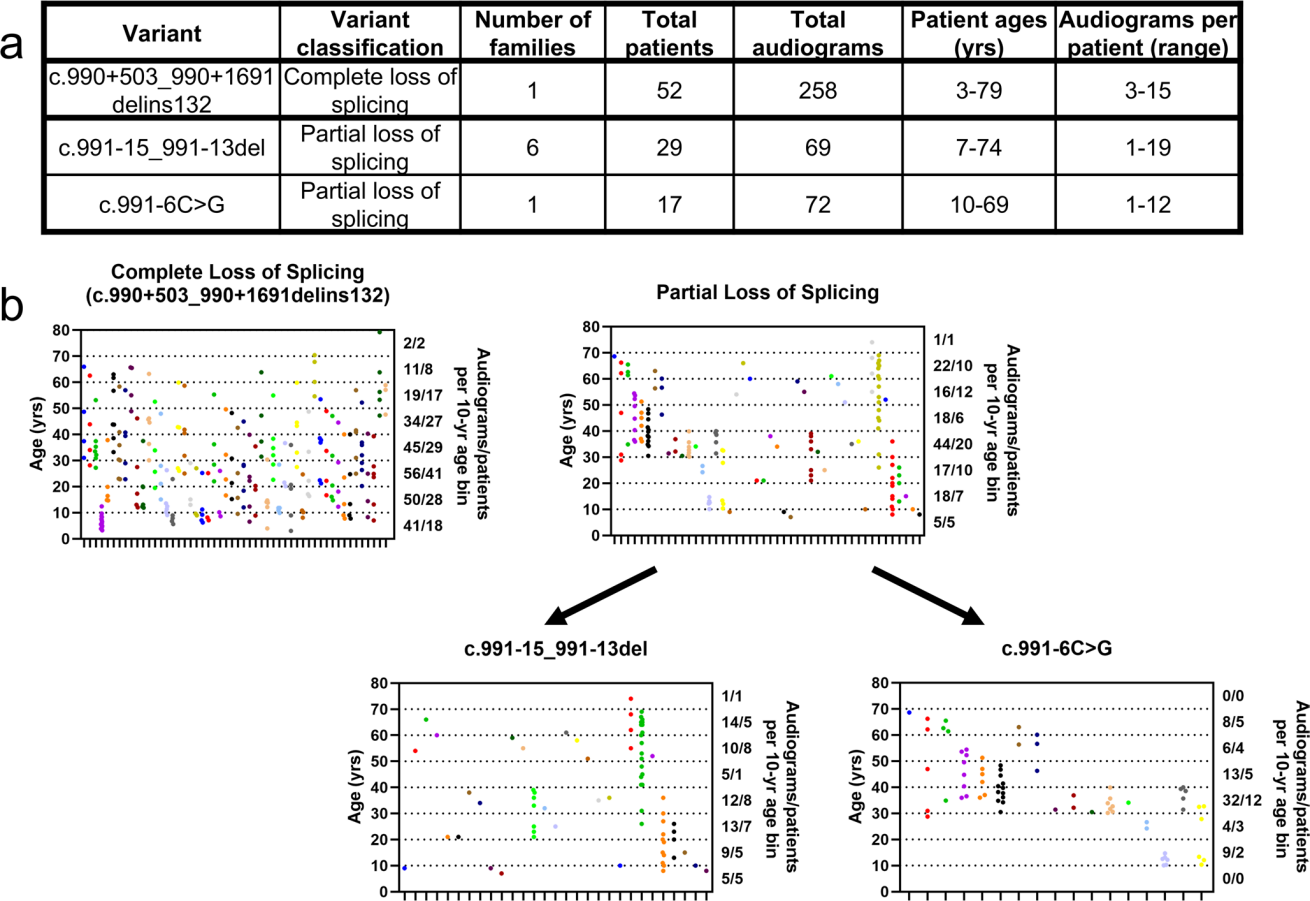
Composition of the dataset used for mixed effects modeling. **a** Summary statistics of the dataset used. **b** Scatter plots showing the per patient distribution of audiograms and the ages when the audiograms were collected. Individual patients are organized by column, as indicated by a hash on the x-axis. Cases of partial loss of splicing can be subdivided into the c.991 − 15_991-13del and c.991–6 C > G variants

A summary of the number of families, unique patients, and audiograms, and the distributions of ages is provided in Fig. 2. The data were analyzed by both individual variant and as cases of complete versus partial loss of splicing. Cases of partial loss of splicing were composed of pooled audiometric data from the c.991–6 C > G and c.991 − 15_991-13del variants.

**Fig. 3:**
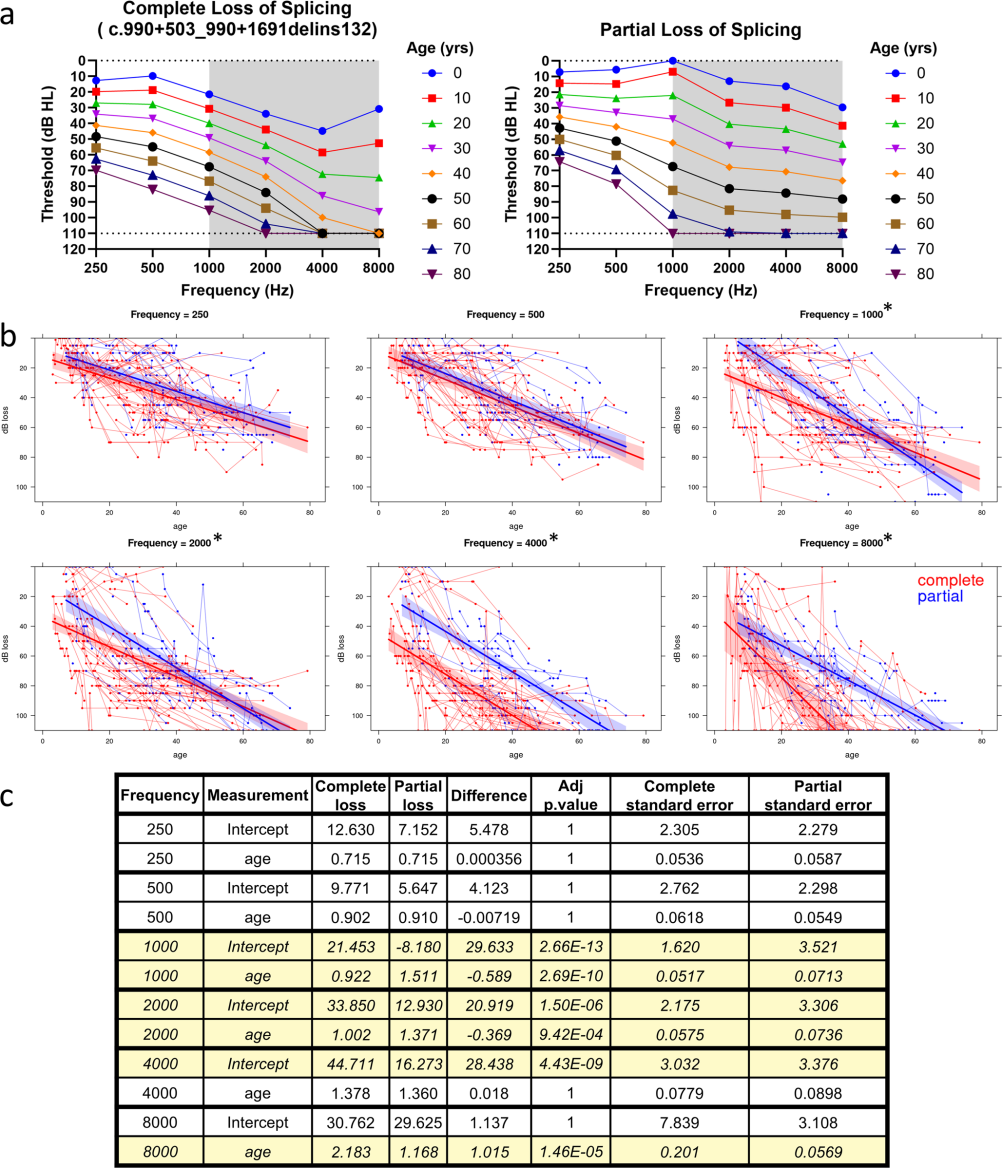
Audiometric analysis. **a** Age-Related Typical Audiograms for cases of complete and partial loss of splicing. Frequencies where there is a statistically significant difference in thresholds are shaded in gray. **b** Individual mixed effects linear modeling by frequency. Dots represent individual audiometric measurements. Connecting lines indicate measurements taken from the same patient. Shaded areas surrounding the regression indicate 95% confidence intervals. Overlapping shaded areas indicate no significant differences at that age. * indicates that there were significant (adjusted p-value < 0.05) differences in the overall thresholds between the groups at the given frequency. **c** Summary table of the mixed effects modeling. Components of the mixed effects modeling that have a statistically significant difference are highlighted

There were statistically significant differences in the rate of progression and/or baseline hearing between the partial and complete loss of splicing groups at the 1000, 2000, 4000, and 8000 Hz frequencies (Fig. 3a). We also found differences between the groups when analyzed by individual variant, including observable differences in the two partial loss-of-splicing variants at the 4000 and 8000 Hz frequencies (Supplementary Fig. S3).

### Assessment of in silico prediction tools

**Fig. 4:**
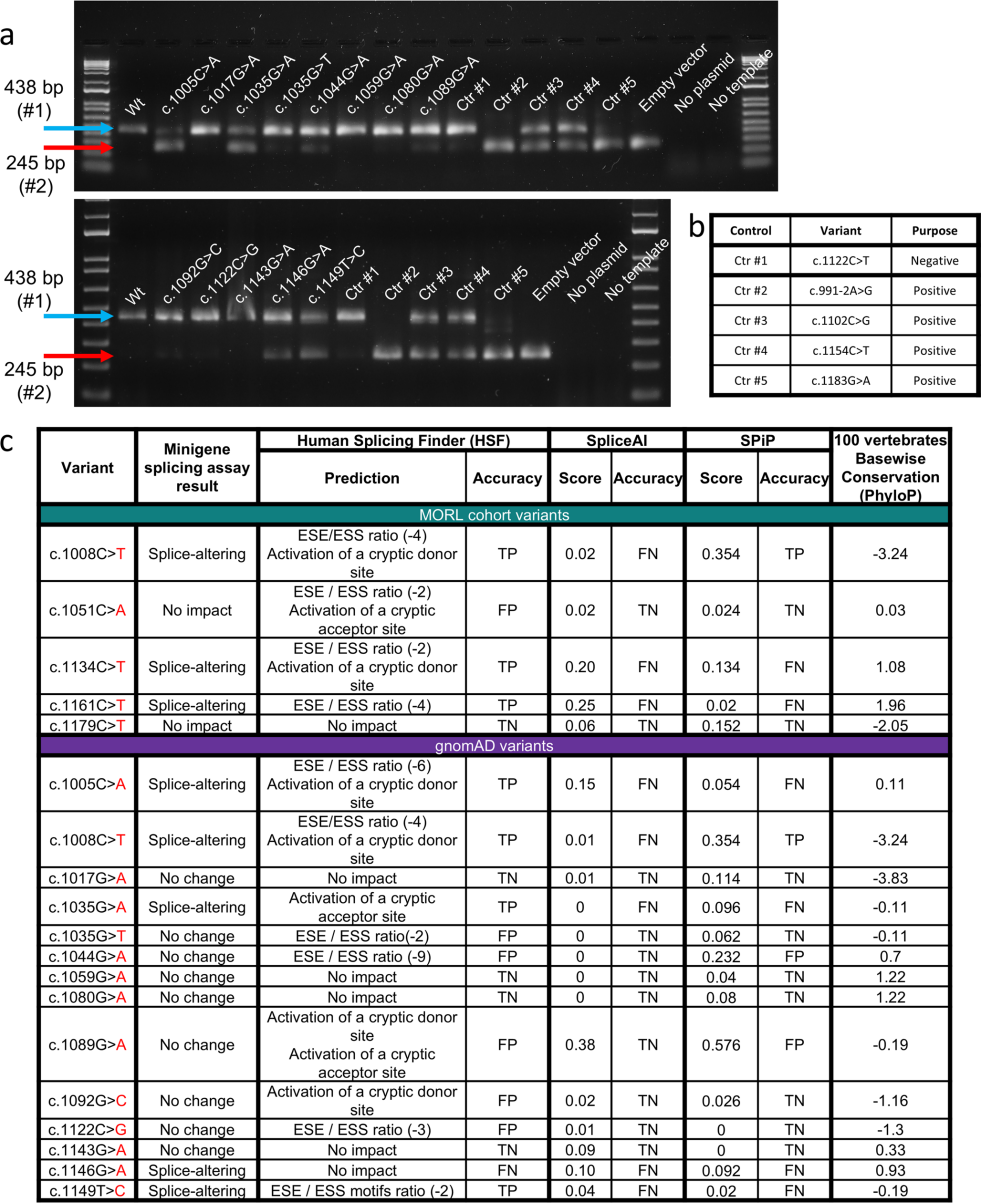
In vitro validation of in silico prediction tools. **a** Minigene splicing assay results visualized on gel electrophoresis for wild-type and mutant constructs of rare synonymous GSDME exon 8 variants in gnomAD. **b** Table describing the controls used, their identities, and functions. **c** Table summarizing the minigene splicing assay results and the corresponding predictions of HSF, SpliceAI, and SPiP in the context of the MORL cohort and rare gnomAD variants. True Positive (TP); True Negative (TN); False Positive (FP); False Negative (FN)

**Fig. 5:**
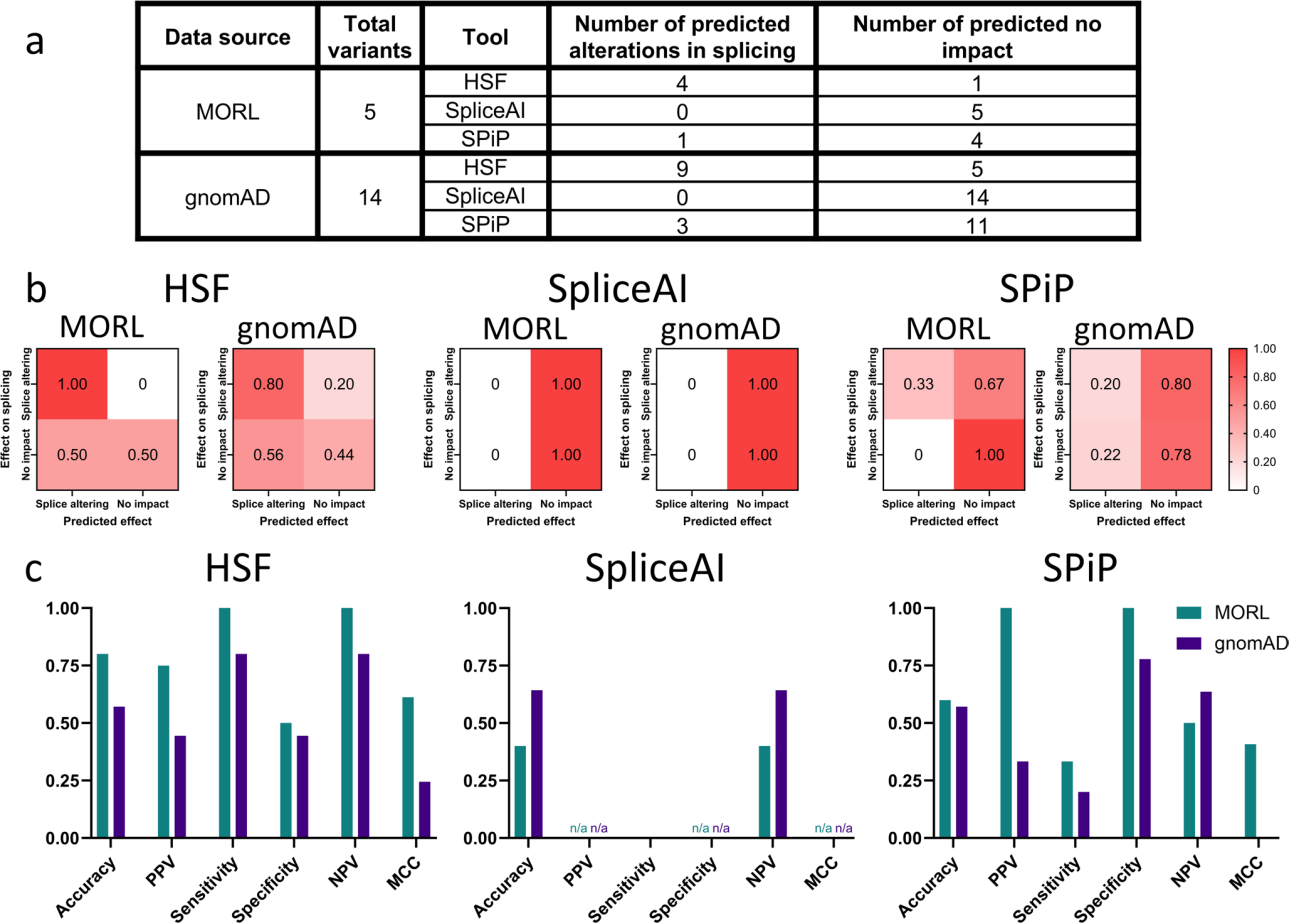
Evaluation of the accuracy of HSF, SpliceAI, and SPiP. **a** Summary statistics of the predictions of HSF, SpliceAI, and SPiP in the context of variants from the MORL and gnomAD. **b** Confusion matrixes comparing the in silico prediction of HSF, SpliceAI, and SPiP versus results from minigene splicing assay. **c** Details about the predictive value. Values that could not be calculated are indicated with “n/a”. Positive Predictive Value (PPV); Negative Predictive Value (NPV); Matthews Correlation Coefficient (MCC)

Four of the 14 variants from gnomAD were shown to alter splicing in a minigene splicing assay (Fig. 4). There was limited correlation between the effect observed on the minigene splicing assay and the in silico prediction from HSF and SPiP for both variants identified in the retrospective MORL and gnomAD cohorts. SpliceAI failed to make any positive predictions of a splice-altering effect for variants in both the retrospective and gnomAD cohorts (Figs. 4 and 5). There was no identifiable link between conservation and loss of splicing in both the retrospective and gnomAD cohorts (*p* = 0.5637 and *p* = 0.8415 respectively).

## Discussion

The pathology of synonymous variants can be complex, with multiple possible causes of pathology such as alterations in splicing, protein stability, and translation (Hunt et al. 2014; Lin et al. 2023). However, in the case of *GSDME*, the gain-of-function mechanism reflects a simpler pathology of exon skipping that generates a transcript that lacks exon 8. This lack of exon 8 results in the formation of a constitutively active protein that results in apoptosis. This pathophysiology has been verified in both cell culture (de Beeck et al. 2012) and mouse models (Xiao et al. 2024). This common pathophysiology allows one to assume that all pathogenic SNVs involve aberrant splicing and thereby eliminates the need to investigate other factors such as changes in mRNA structure and stability, miRNA binding, and codon usage.

These results are consistent with our hypothesis that there are pathogenic synonymous variants that were otherwise being filtered out. Previously, all known pathogenic *GSDME* variants were in the intronic regions flanking exon 8, missense variants within exon 8, or structural variants. Our identification of the first pathogenic synonymous *GSDME* variants illustrates the importance of assessing synonymous variants in the context of genetic diagnostics. Consistent with previous work (Booth et al. 2018), we also observe splice-altering variants outside the proximity of the exon boundaries (Fig. 1e, Supplementary Fig. S4). These results highlight the need to assess variants for alterations in splicing regardless of their location within the exon and the need for in silico prediction tools that are not constrained to the proximity of splice acceptor and donor sites.

The recurrence of the c.1161 C > T, p.(Tyr387Tyr) variant in two unrelated probands is not entirely unexpected (Fig. 1a). Despite the relative rarity of *GSDME* as a cause of HL (< 1% of genetically diagnosed cases) (Sloan-Heggen et al. 2016), there are other recurring pathogenic variants such as the c.991–2 A > G (Booth et al. 2018) and the c.991 − 15_991-13del (Booth et al. 2020) variants. Furthermore, the polypyrimidine tract of *GSDME* intron 7 is a mutational hotspot (Booth et al. 2020), which raises the possibility of the existence of other mutational hotspots in the same gene. This presence of rare, but recurring variants indicates the need for functional assessment of variants regardless of their rarity.

Our observations of varying levels of aberrant splicing in minigene is not unexpected given that it has already been reported that the c.991–6 C > G variant results in less of the truncated transcript compared to the c.990 + 503_990 + 1691delins132 variant (Bischoff et al. 2004). Many genes implicated in HL have genotype-phenotype correlations and/or population-level differences (Walls et al. 2020). It is logical to hypothesize that greater levels of aberrant splicing will corelate with greater levels of mutant protein, and by extension more hair cell death and more severe HL. Audiometric analysis supports this hypothesis. Complete loss of splicing results in more severe HL compared to partial loss of splicing, as reflected in more severe HL at a given age and/or more rapid progression at the middle and high frequencies (Fig. 2). The lack of statistically significant differences in the severity of HL at the low frequencies may be due to the lack of statistical power. The HL is mild at the low frequencies (Thorpe et al. 2022) and the differences at 250 and 500 Hz are very small (Fig. 2b-c).

While some HL genes such as *TECTA* and *WFS1* have ethnic-based differences (Walls et al. 2020), they are not observed for *GSDME*. The c.991–6 C > G and c.990 + 503_990 + 1691delins132 variants were both observed in large Dutch families (Bischoff et al. 2004) and demonstrate results consistent with complete loss of splicing causing more severe HL (Supplementary Fig. S3). These observations also are consistent with the report that persons carrying the c.991–6 C > G variant have better speech recognition than persons carrying the c.990 + 503_990 + 1691delins132 variant (Bischoff et al. 2004) and are consistent with a prior investigation of the *GSDME* c.991 − 15_991-13del variant that was unable to find ethnic-based HL variability (Booth et al. 2020). Taken together, these findings suggest that the primary driver of differences in the severity of *GSDME*-related HL is a variant-specific effect.

We also observe that of the three *GSDME* variants analyzed for genotype-phenotype correlations, the c.991 − 15_991-13del variant has the mildest HL (Supplementary Fig. S3). Presumably, this would indicate that the c.991 − 15_991-13del variant has the least amount of aberrant splicing. Future work that evaluates the amount of aberrant splicing in a uniform manner could confirm this hypothesis. A detailed comparison of the precise levels of aberrant splicing would be of future interest because it could lead to the determination of the minimum amount of aberrant splicing required to cause HL and thereby guide potential therapeutic interventions such as knockdown of the mutant transcript.

In the context of *GSDME*-related HL, in silico prediction tools should be used cautiously. HSF, SpliceAI and SPiP in the context of the retrospective MORL and gnomAD cohorts are highly inaccurate (Figs. 4 and 5). This conclusion is consistent with other reports recommending in silico assessment to be used as a screening tool rather than as a diagnostic tool (Soukarieh et al. 2016; Moles-Fernández et al. 2018; Katneni et al. 2019; Walker et al. 2023; Sullivan et al. 2025). We do point out that HSF might be useful as a screening tool for splice-altering variants because it has a sensitivity of over 75% in the context of *GSDME*. In contrast, the utility of SpliceAI appears to be much more limited (Fig. 5).

We do note the reported accuracy in our cohorts is lower than that reported in other studies. This difference in accuracy may reflect the specific nature of *GSDME*, where the pathology excludes certain types of aberrant splicing such as the formation of cryptic splice acceptor and donor sites that are seen in other genetic disorders. The possibility of a gene-specific difference is not surprising, given that there are other deafness gene-specific differences such as in minor allele frequency thresholds (Azaiez et al. 2018). In addition, we investigated variants regardless of their location within an exon. In contrast, most studies and prediction tools prioritize investigating variants near the canonical splice donor and acceptor sites (Dionnet et al. 2020; Walker et al. 2023; Sullivan et al. 2025). Our observations of splice-altering variants outside the splicing consensus region (Supplementary Fig. S4) also prevented us from using commonly available and user friendly splice prediction tools such as MaxEntScan (Yeo and Burge 2004), dbscSNV (Jian et al. 2014), and SpliceAPP (Huang et al. 2024) because they lacked the ability to assess the majority of variants in our cohorts. These results indicate the need for *in silico* prediction tools that can assess variants for splice-altering effects regardless of their location in the genome.

The possibility of a bias due to a focus on variants near the canonical splice acceptor and donor sites would be consistent with HSF, SpliceAI, and SPiP appearing to be more accurate when predicting the impact of previously reported pathogenic variants (Supplementary Table 5). This bias presumably could be explained by the fact that both HSF, SpliceAI and SPiP are trained primarily on the normal splicing patterns observed with wild-type exons (Desmet et al. 2009; Jaganathan et al. 2019; Leman et al. 2022). However, one must be cautious when drawing conclusions from a retrospective assessment because of publication bias and data circularity. For example, the three missense variants were already flagged and prioritized for investigation by HSF at the time of their publication (Booth et al. 2018). It is also important to note that both HSF and SpliceAI have a false negative prediction for the c.991–6 C > G variant (Supplementary Table 5), despite the presence of extensive genetic evidence as well as harvested RNA to show that it is a pathogenic, splice-altering variant (Bischoff et al. 2004). We also highlight that in the context of the MORL cohort, a reported family history consistent with autosomal dominant HL had a stronger correlation with altered splicing than the HSF and SpliceAI predictions (Figs. 1a-b and 4b). These results indicate that in silico assessment cannot be used to override genetic evidence.

Previous work suggested using conservation as a way of prioritizing variants for their impact on splicing in the context of *GSDME* missense variants (Booth et al. 2018). Our results indicate that in the context of synonymous and intronic variants, there is no observable correlation between conservation and a variant’s impact on splicing (Fig. 4b and Supplementary Table 3). As a result, one should be cautious when using conservation as a method of screening for splice-altering variants.

In conclusion, the assessment of synonymous variants is an important part of genetic diagnostics. We expand the mutational landscape of *GSDME*-related HL to include synonymous variants. Given that there were 3 missense variants previously identified in the MORL cohort (Booth et al. 2018) and 3 synonymous variants identified in this study of the MORL cohort, we show that there is a near even split between pathogenic missense and synonymous *GSDME* variants. We also demonstrate that there is a recurring pathogenic synonymous variant. Finally, we identify that there are variant-dependent differences in aberrant splicing levels. These differences in aberrant splicing are reflected in the phenotype-genotype correlation of *GSDME*-related HL, where greater amounts of expressed mutant protein result in more severe and rapidly progressing HL in the middle to high frequencies. These differences in HL suggest that a knockdown strategy targeting the mutant transcript can reduce the impact of *GSDME*-related HL.

Identification of pathogenic variants remains a challenge, particularly when one is interested in identifying splice-altering variants that reside outside of the proximity of the canonical splice sites. One must consider the potential impact on splicing regardless of the variant’s in silico prediction, location within the exon, or conservation.

## Supplementary Information

Below is the link to the electronic supplementary material.

Supplementary Material 1

Supplementary Material 2

Supplementary Material 3

## Data Availability

The code for the audiometric analysis is available in Zenodo with the identifier [https://doi.org/10.5281/zenodo.15284253](https:/doi.org/10.5281/zenodo.15284253) . Other data and code supporting the findings of this study are available from the corresponding author upon reasonable request.
