# Supplementary material for: Investigation of GSDME results in the identification of the first pathogenic synonymous variants and genotype-phenotype correlations
Source: Hum Genet. 2025 Sep 29;144(11-12):1127–38. doi: 10.1007/s00439-025-02782-6 (PMC12689683; doi:10.1007/s00439-025-02782-6)
Supplement: Supplementary file 3 — Supplementary Material 3 [file 439_2025_2782_MOESM3_ESM.docx]

# **Supplementary Information**

**Supplementary Fig. S1** Pure-tone audiograms of the patients with causative synonymous *GSDME* variants

**Supplementary Fig. S2** Chromatograms showing available Sanger sequencing and segregation analysis of newly identified *GSDME* families

**Supplementary Fig. S3** Audiometric analysis by variant (a) Age-Related Typical Audiograms. Frequencies where there is a statistically significant difference in thresholds are shaded in gray. (b) Individual mixed effects linear modeling by frequency. Dots represent individual audiometric measurements. Connecting lines indicate measurements taken from the same patient. Shaded areas surrounding the regression indicate 95% confidence intervals. Overlapping shaded areas indicate no significant differences at that age. * indicates that there were significant (adjusted p-value < 0.05) differences in the overall thresholds between the groups at the given frequency. (c) Summary table of the linear mixed effects modeling. Components of the linear modeling that have a statistically significant difference are highlighted.

**Supplementary Fig. S4** Precise locations of known splice-altering *GSDME* variants based on the literature and from this work.

**Supplementary Table 1** Table of all high quality variants with MAF < 1% that were considered in the differential diagnosis of the *GSDME* patients in the MORL cohort. ND, not detected.

**Supplementary Table 2** Table of population specific MAF for all *GSDME* variants in this study that were evaluated with a minigene splicing assay.

**Supplementary Table 3** Table of pathogenic *GSDME* variants that were reviewed for potential inclusion for genotype phenotype correlations. Variants that were included in the genotype-phenotype correlation are highlighted in gray.

**Supplementary Table 4** Table of densitometry measurements for *GSDME* variants in the MORL cohort that were evaluated with a minigene splicing assay.

**Supplementary Table 5** Retrospective assessment of the accuracy of HSF, SpliceAI, and SPiP in the context of previously reported pathogenic *GSDME* variants. TP, true positive; TN, true negative; FP, false positive; FN, false negative.

**Supplementary Fig. S1**

**
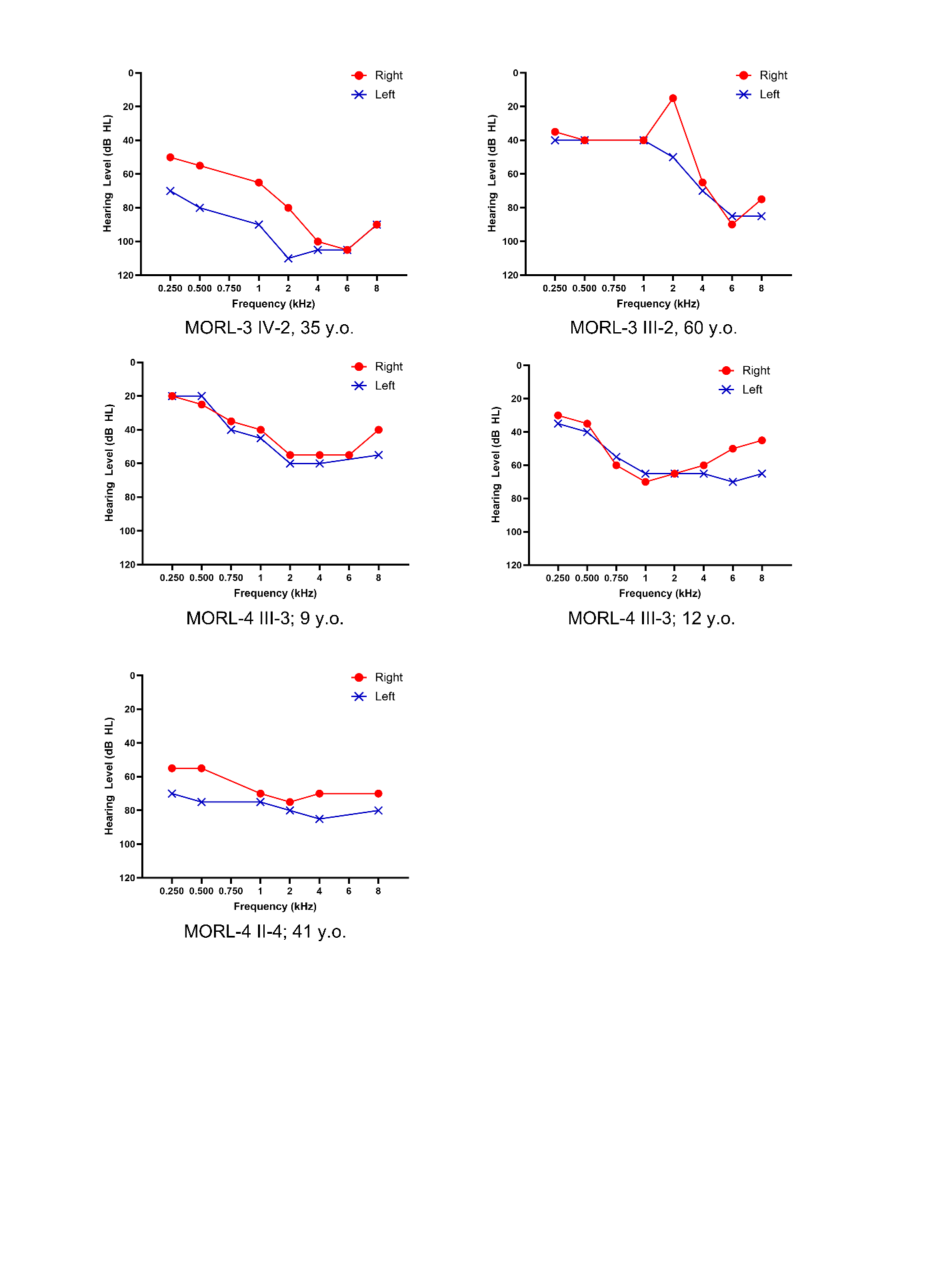
**

**Supplementary Fig. S2**

**
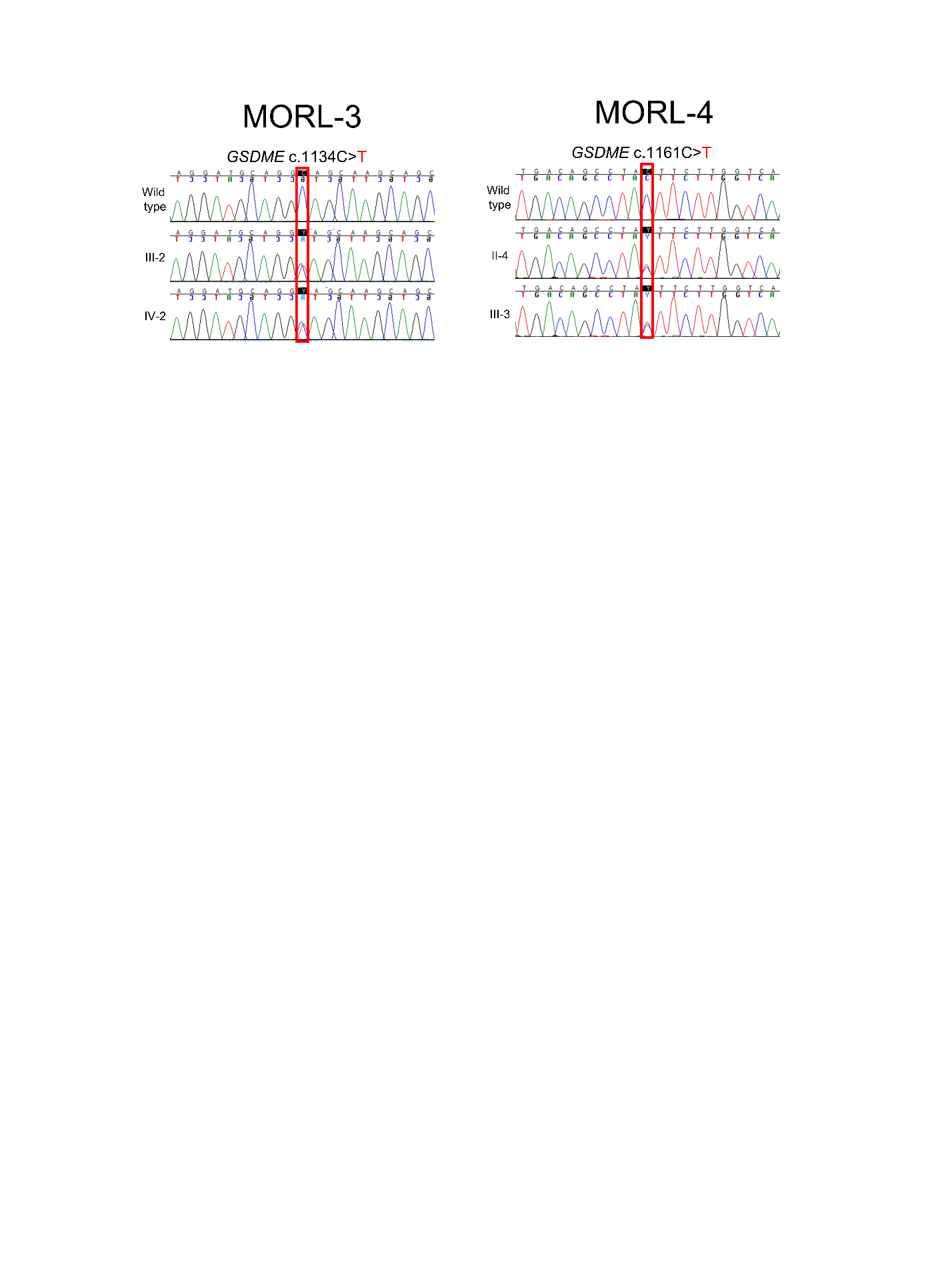
**

**Supplementary Fig. S3**

**
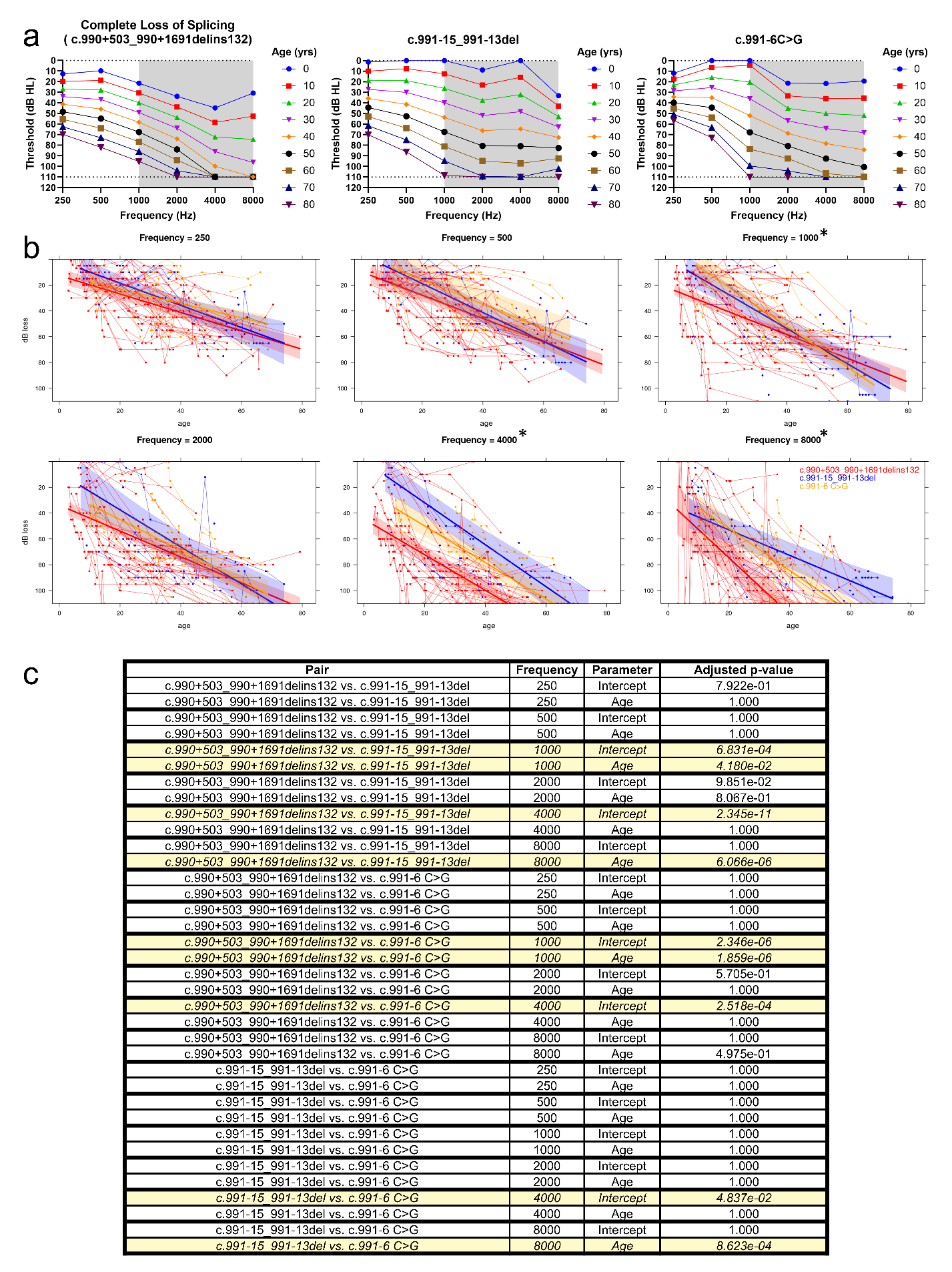
**

**Supplementary Fig. S4**

**
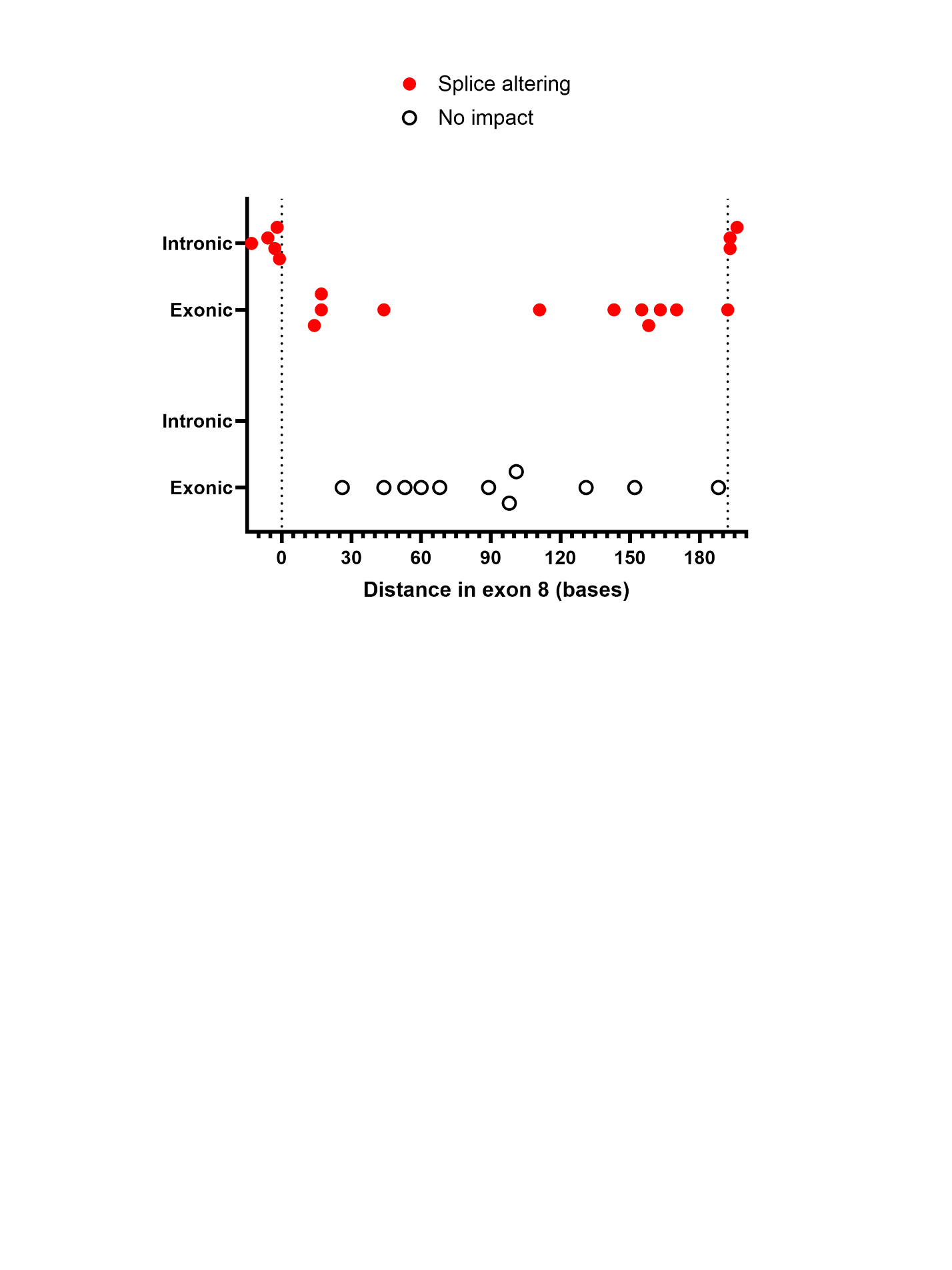
**

**Supplementary Table 1:**

[This table is provided as a .xlxs spreadsheet]

**Supplementary Table 2:**

[This table is provided as a .xlxs spreadsheet]

**Supplementary Table 3:**

| **Variant** | **Degree of Splice Impact** | **Citation for splicing analysis** | **Reported families** | **Total patients** | **Total audiograms** | **Patient ages (range, yrs)** | **Audiograms per patient (range)** |
| --- | --- | --- | --- | --- | --- | --- | --- |
| c.[990+793_1007del; 1029_1183+1376del] | Complete  (Presumed) | Mansard et al., 2022 | 1 | 1 | 1 | 30 | 1 |
| *c.990+503_990+1691delins132* | *Complete* | *Bischoff et al., 2004* | *1* | *52* | *258* | *3-79* | *3-15* |
| c.991-60_991-1095del | Unknown | Mansard et al., 2022 | 1 | 8 | 8 | 4-59 | 1 |
| *c.991-15_991-13del* | *Partial* | *Yu et al., 2003* | *6* | *29* | *69* | *7-74* | *1-19* |
| *c.991-6C>G* | *Partial* | *Bischoff et al., 2004* | *1* | *17* | *72* | *10-69* | *1-12* |
| c.991-3C>A | Unknown | Wang et al., 2018 | 1 | 1 | 1 | 36 | 1 |
| c.991-2A>G | Complete | This paper | 2 | 22 | 7 | 28-34 | 1-2 |
| c.991-1G>C | Unknown | Chen et al., 2020 | 1 | 2 | 2 | 30-55 | 1 |
| c.1008C>T | Partial | This paper | 1 | 1 | 0 | N/A | N/A |
| c.1102C>G | Partial | This paper | 1 | 1 | 1 | 33 | 1 |
| c.1134C>T | Partial | This paper | 1 | 2 | 2 | 35-60 | 1 |
| c.1154C>G | Partial | This paper | 1 | 3 | 3 | 33-51 | 1 |
| c.1161C>T | Partial | This paper | 1 | 2 | 4 | 9-41 | 1-2 |
| c.1183G>A | Complete | This paper | 1 | 2 | 2 | 28-34 | 1 |
| c.1183+1G>C | Complete | Li et al., 2022 | 1 | 2 | 3 | 30-67 | 1-2 |
| c.1183+1del | Unknown | Li Yang et al., 2015 | 1 | 2 | 2 | 24-55 | 1 |
| c.1183+4A>G | Unknown | Cheng et al., 2007 | 1 | 4 | 4 | 15-70 | 1 |

**Supplementary Table 4**:

| Variant | With exon 8 (%) | Without exon 8 (%) | Std. error |
| --- | --- | --- | --- |
| Wt | 95.0% | 5.0% | 0.010589 |
| c.1008C>T | 56.2% | 43.8% | 0.017175 |
| c.1051C>A | 83.0% | 17.0% | 0.007551 |
| c.1134C>T | 53.5% | 46.5% | 0.040432 |
| c.1161C>T | 48.4% | 51.6% | 0.00935 |
| c.1179C>T | 82.0% | 18.0% | 0.056105 |
| Ctr #1 | 91.0% | 9.0% | 0.001388 |
| Ctr #2 | 5.4% | 94.6% | 0.015359 |
| Ctr #3 | 47.1% | 52.9% | 0.014795 |
| Ctr #4 | 55.4% | 44.6% | 0.020672 |
| Ctr #5 | 10.6% | 89.4% | 0.009502 |
| EV | 3.0% | 97.0% | 0.016378 |

**Supplementary Table 5**:

| **Variant** | **Impact on splicing** | **Human Splicing Finder (HSF)** | | **SpliceAI** | | **SPiP** | | **100 vertebrates Basewise Conservation (PhyloP)** |
| --- | --- | --- | --- | --- | --- | --- | --- | --- |
|  |  | **Prediction** | **Accuracy** | **Score** | **Accuracy** | **Score** | **Accuracy** |  |
| Previously reported variants | | | | | | | | |
| c.991-15_991-13del | Splice-altering | Activation of cryptic donor site  Broken WT acceptor site  Activation of cryptic acceptor site | TP | 0.02 | FN | 0 | FN | 0.26 |
| c.991-6C>G | Splice-altering | No impact | FN | 0.03 | FN | 0.098 | TP | -0.11 |
| c.991-3C>A | Splice-altering | Broken WT acceptor site | TP | 0.06 | FN | 0.99 | TP | 2.27 |
| c.991-2A>G | Splice-altering | Broken WT acceptor site  Activation of cryptic donor site | TP | 1 | TP | 1 | TP | 3.83 |
| c.991-1G>C | Splice-altering | Broken WT acceptor site | TP | 0.99 | TP | 1 | TP | 3.75 |
| c.1102C>G | Splice-altering | ESE / ESS motifs ratio (-5)  Activation of cryptic acceptor site | TP | 0.02 | FN | 0.076 | FN | 1.37 |
| c.1154C>G | Splice-altering | ESE / ESS motifs ratio (-5)  Activation of cryptic acceptor site | TP | 0.22 | FN | 0.1 | FN | 2.79 |
| c.1183G>A | Splice-altering | Broken WT donor site | TP | 0.71 | TP | 0.868 | TP | 2.71 |
| c.1183+1G>C | Splice-altering | Broken WT donor site | TP | 1 | TP | 1 | TP | 4.13 |
| c.1183+1del | Splice-altering | Broken WT donor site | TP | 1 | TP | 1 | TP | 4.13 |
| c.1183+4A>G | Splice-altering | Broken WT donor site  Activation of cryptic acceptor site | TP | 0.57 | TP | 0.884 | TP | -0.49 |
